# One-step heating strategy for efficient solubilization of recombinant spider silk protein from inclusion bodies

**DOI:** 10.1186/s12896-020-00630-1

**Published:** 2020-07-10

**Authors:** Hui Cai, Gefei Chen, Hairui Yu, Ying Tang, Sidong Xiong, Xingmei Qi

**Affiliations:** 1 The Jiangsu Key Laboratory of Infection and Immunity, Institutes of Biology and Medical Sciences, Soochow University, Suzhou, 215123 Jiangsu China; 2 Department of Neurobiology, Care Sciences and Society, Center for Alzheimer Research, Division of Neurogeriatrics, Karolinska Institutet, 14157 Huddinge, Sweden

**Keywords:** Heating, Recombinant spidroin, Inclusion body solubilization, NM IBs

## Abstract

**Background:**

Spider silk is a proteinaceous fiber with remarkable mechanical properties spun from spider silk proteins (spidroins). Engineering spidroins have been successfully produced in a variety of heterologous hosts and the most widely used expression system is *Escherichia coli* (*E. coli*). So far, recombinantly expressed spidroins often form insoluble inclusion bodies (IBs), which will often be dissolved under extremely harsh conditions in a traditional manner, e.g. either 8 mol/L urea or 6 mol/L guanidine hydrochloride, highly risking to poor recovery of bioactive proteins as well as unexpected precipitations during dialysis process.

**Results:**

Here, we present a mild solubilization strategy—one-step heating method to solubilize spidroins from IBs, with combining spidroins’ high thermal stability with low concentration of urea. A 430-aa recombinant protein (designated as NM) derived from the minor ampullate spidroin of *Araneus ventricosus* was expressed in *E. coli*, and the recombinant proteins were mainly present in insoluble fraction as IBs. The isolated IBs were solubilized parallelly by both traditional urea-denatured method and one-step heating method, respectively. The solubilization efficiency of NM IBs in Tris-HCl pH 8.0 containing 4 mol/L urea by one-step heating method was already comparable to that of 7 mol/L urea with using traditional urea-denatured method. The effects of buffer, pH and temperature conditions on NM IBs solubilization of one-step heating method were evaluated, respectively, based on which the recommended conditions are: heating temperature 70–90 °C for 20 min, pH 7.0–10, urea concentration 2–4 mol/L in normal biological buffers. The recombinant NM generated via the one-step heating method held the potential functions with self-assembling into sphere nanoparticles with smooth morphology.

**Conclusions:**

The one-step heating method introduced here efficiently solubilizes IBs under relatively mild conditions compared to the traditional ones, which might be important for the downstream applications; however, this protocol should be pursued carefully in terms of urea-induced modification sensitive applications. Further, this method can be applied under broad buffer, pH and temperature conditions, conferring the potential to apply to other thermal stable proteins.

## Background

Spider silk is a kind of ideal biomaterial attributed to its extraordinary performances, such as outstanding mechanical properties and excellent biocompatibility [1–3]. In nature silk proteins are specifically spun into fibers, whereas after processed under various condition in vitro they can assemble into different forms with distinct morphologies, e.g. films, hydrogels, fibers, capsules and particles [2, 4]. The versatility of silk proteins along with their biochemical properties make silk-derived materials potentially suitable for tissue engineering and regeneration as well as controllable delivery of protein drugs and peptide vaccines [5–9]. Due to difficulties in breeding spiders and collecting silks, native spider silk is not economic and realistic to harvest on a large scale, while in nowadays recombinant production is becoming the main strategy for obtaining either spider silk protein (spidroin) or artificial spider fiber [10–13]. Orb-weaving spiders can have up to seven different silk glands for manufacturing different types of silk with specific biological function and unique mechanical properties [14]. Most recent studies have focused on the dragline silk, which is displayed with high tensile strength. The recombinant truncated dragline silk protein (major ampullate spidroin, MaSp) was shown to form nanoparticles with possibilities as drug delivery vehicles and peptide vaccines delivery system [4, 8, 9]. However, alternative biomaterials with different properties may be needed for different applications. The minor ampullate silk, distinctive from dragline silk, is used for prey wrapping and web-stabilizing auxiliary spirals [15]. Interestingly, this silk shares similar tensile strength as dragline silk but with low elasticity, and does not supercontract when hydrated [16]. Hence, minor ampullate silk could be interesting for particular biomedical applications.

Spider silk is built up of large spidroins consisting of three parts, a non-repetitive N-terminal (NT) domain, a predominant highly repetitive central domain, and a non-repetitive C-terminal (CT) domain [15, 17, 18]. For heterologous expression, the repetitive gene sequence coding for the repetitive central domain usually causes problems such as premature termination in protein synthesis, low yield and poor solubility. Several strategies have been tried to overcome these problems, including codon optimization, growing in enriched media and use of different gene constructs [19–21]. Additionally, a variety of heterologous expression hosts have also been attempted to produce recombinant spidroins, e.g. bacteria, yeast, plants, mammalian cells, and transgenic animals, each with its own pros and cons in terms of cost, manipulation, expression levels and contaminations [22–24]. The most widely used expression system is *Escherichia coli* (*E. coli*) owing to the most efficient, simple manipulation and cost-efficient production suitable for large-scale production [20, 22, 25].

To prepare pure recombinant spidroins, different strategies, e.g. affinity chromatography [26], thermal extraction [27, 28] and acidic extraction [27–29] have been pursued. Affinity chromatography is the most often used approach for recombinant spidroin purification, though an affinity tag is required. As the affinity tag might alter the protein properties, an additional step is normally necessary to cleave it out. Thermal and acidic extraction methods are based on spidroins’ innate properties—thermal stability and solubility characteristics, during which the majority of the host proteins are precipitated at high temperature and concentrated acid, while silk proteins remain soluble. These methods circumvent the need of an affinity tag, which is favorable for biomedical applications. Unfortunately, the pure protein from the thermal extraction method is usually largely diluted and an ammonium sulfated mediated concentration step is needed, which often induces precipitation [27, 28]. To obtain soluble proteins, the precipitated proteins are dissolved in guanidine and dialyzed against non-denaturing buffer. Proteins extracted in presence of organic acids normally have to pass an affinity or ion exchange column, which is a long-lasting time and high cost process [29]. Besides, expression of recombinant spidroins has often resulted in insoluble IB formation [24, 30], however, in some cases forming of IBs is advantageous as IBs are easily isolated with high yield and purity. Traditionally, protein solubilization from IBs is often achieved under harsh conditions, i.e. using high concentration of denaturant—8 mol/L urea or 6 mol/L guanidine hydrochloride. Before processing the target proteins into various formats, these harsh denaturants must be removed completely. Generally, solubilization of IBs with high concentration of denaturing reagents often leads to poor recovery of bioactive species, and large amount of precipitations form during the refolding process [31, 32].

In this study, to make efficient preparation of recombinant spidroins and give some insights for assembly characterization, we first expressed a 430 residues truncated spidroin (designated as NM) derived from *Araneus ventricosus* minor ampullate spidroin (MiSp) in *E. coli*. This truncated spidroin consists of the conserved nonrepetitive NT domain and one repetitive unit, while the full-length *A. ventricosus* MiSp comprises a central predominant repetitive region and the conserved nonrepetitive NT and CT domains [15]. The recombinant NM was successfully expressed in *E. coli* but as IBs. We then evaluated a relatively mild strategy— solubilizing NM IBs with short-term heating in the presence of low concentration of urea, referred as one-step heating method. With this method, we got highly pure and concentrated NM proteins, capable of self-assembling into sphere nanoparticles.

## Results

### NM architecture and recombinant expression

**Fig. 1:**
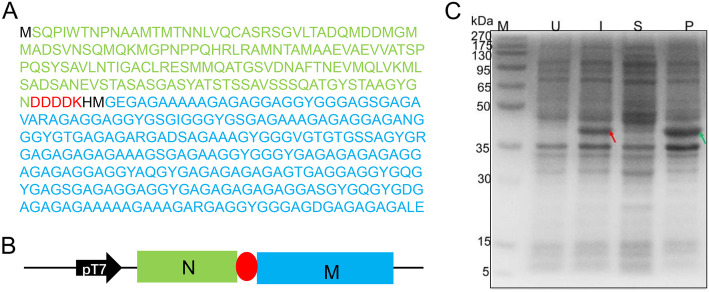
Amino acid sequence, plasmid construction and NM expression. **a** Amino acid sequence of NM protein derived from the *A. ventricosus* MiSp**.** The NM sequence consists of the NT domain (161-aa, in green) and a truncated repetitive region (261-aa, in blue), between which there is an enterokinase cleavage site (DDDDK). **b** The gene fragment coding for NM was inserted into pET-32a plasmid within *Nde*I and *Xho*I restriction sites to construct expression vector pET-NM. **c** SDS-PAGE analysis of the recombinant protein NM expressed in *E. coli* BL21 (DE3) cells. Lane M for protein size markers (kDa), lanes U and I for total cellular proteins of *E. coli* before and after IPTG-induced protein expression, respectively, lanes S and P for the supernatants and pellets of the bacterial cell lysate after sonication, respectively. The theoretical molecular weight of NM is 38 kDa

The detailed amino acid sequence of NM is shown in Fig. 1a, while Fig. 1b presents the architecture. The NM recombinant expression was conducted at 37 °C and the final IPTG concentration was 1 mmol/L. Indicated by the SDS-PAGE (Fig. 1c**)**, compared to the control sample (lane U) there is an additional strong band (red arrow) after IPTG induction (lane I), which fits very well to the target protein NM judged by its apparent size of 38 kDa. After bacterial cell disruption, the soluble (lane S) and insoluble cell fractions (lane P) were separated by centrifuge. The NM was mainly in pellet fraction as inclusion bodies (green arrow). These results indicate that recombinant spider silk protein NM can be efficient expressed as inclusion bodies (NM IBs) in *E. coli.*

### Isolation and solubilization of NM IBs

**Fig. 2:**
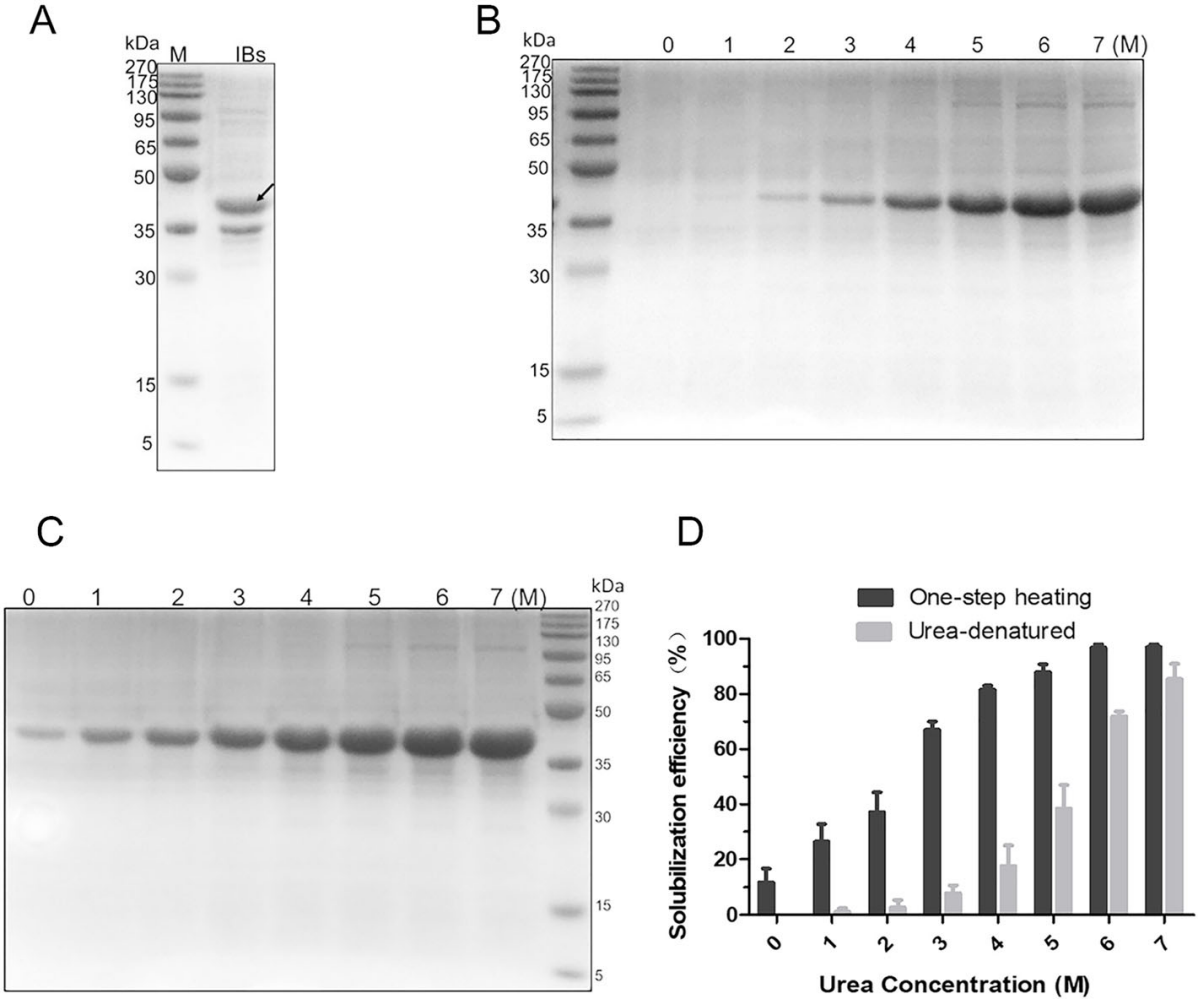
NM IBs isolation and solubilization. **a** SDS-PAGE analysis of the purified NM IBs after extensive washing. **b** SDS-PAGE analysis of the solubilized NM from IBs by traditional urea-denatured method with 0–7 mol/L urea. **c** SDS-PAGE analysis of the solubilized NM protein from IBs by one-step heating method with 0–7 mol/L urea. **d** The protein solubilization efficiency by two different methods with 0–7 mol/L urea

Pure NM IBs were prepared through extensive washing with detergent containing buffer, after which majority of contaminants were removed. The NM IBs purity was evaluated by SDS-PAGE, which is up to 70% (black arrow, Fig. 2a). The purified NM IBs (10 mg/mL wet weight concentration) were resuspended in Tris-HCl pH 8.0 containing different concentrations of urea (0–7 mol/L), and further proceeded with one step-heating method and traditional urea-denature method. The solubilized supernatants of NM IBs from both methods were analyzed by SDS-PAGE (Fig. 2b and c), and the protein concentrations were measured by Micro BCA Protein Assay kit (Table 1), respectively. With traditional urea-denatured method, NM IBs were largely solubilized in Tris-HCl pH 8.0 containing 5–7 mol/L urea, whereas with 0–4 mol/L urea the solubilization efficiency was rather low (Fig. 2b). Interestingly, by performing the one-step heating method Tris-HCl pH 8.0 containing 0–7 mol/L urea all solubilized NM IBs, and at 4 mol/L urea concentration the solubilization efficiency was already up to 80%, which is around three times higher than that of traditional urea-denature method. Interestingly, Tris-HCl pH 8.0 alone could also solubilize NM IBs (Lane 0, Fig. 2c) to some extent. These results indicated that short-term heating improves the solubilization of NM IBs, and the efficiency achieved through one-step heating method in the presence of 4 mol/L urea is comparable to that of 7 mol/L urea via traditional urea-denatured method (Fig. 2d). Hence, for testing other potential affecting factors below, the urea concentration was stuck to 4 mol/L.

**Table 1:** Solubilization of NM spidroins from IBs with different concentrations of urea through two different methods

Urea conc. (mol/L)	Protein concentration (ng/ml)	
	One-step heating method	Traditional urea-denatured
0	483	0
1	952	58
2	1290	157
3	1866	311
4	2335	728
5	2641	1368
6	2786	2143
7	2912	2458

### Solubilization of NM IBs at different temperatures and pHs

**Fig. 3:**
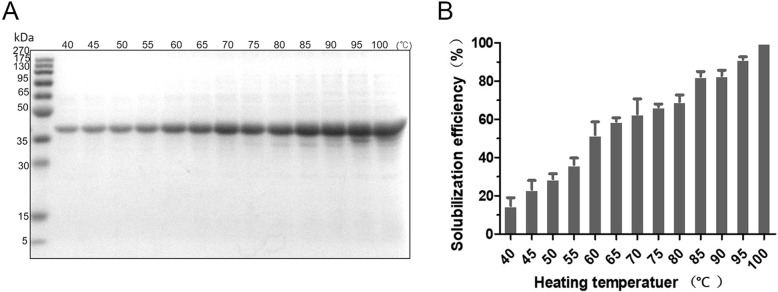
Effects of heating temperature on the solubilization of NM IBs with one-step heating method. Equal amount of purified NM IBs was suspended in 1 mL 10 mmol/L Tris-HCl at pH 8.0 containing 4 mmol/L urea and heating at different temperatures (40–100 °C). **a** SDS-PAGE analysis of the effect of heating temperature. **b** The quantification of solubilization efficiency at different heating temperature

**Fig. 4:**
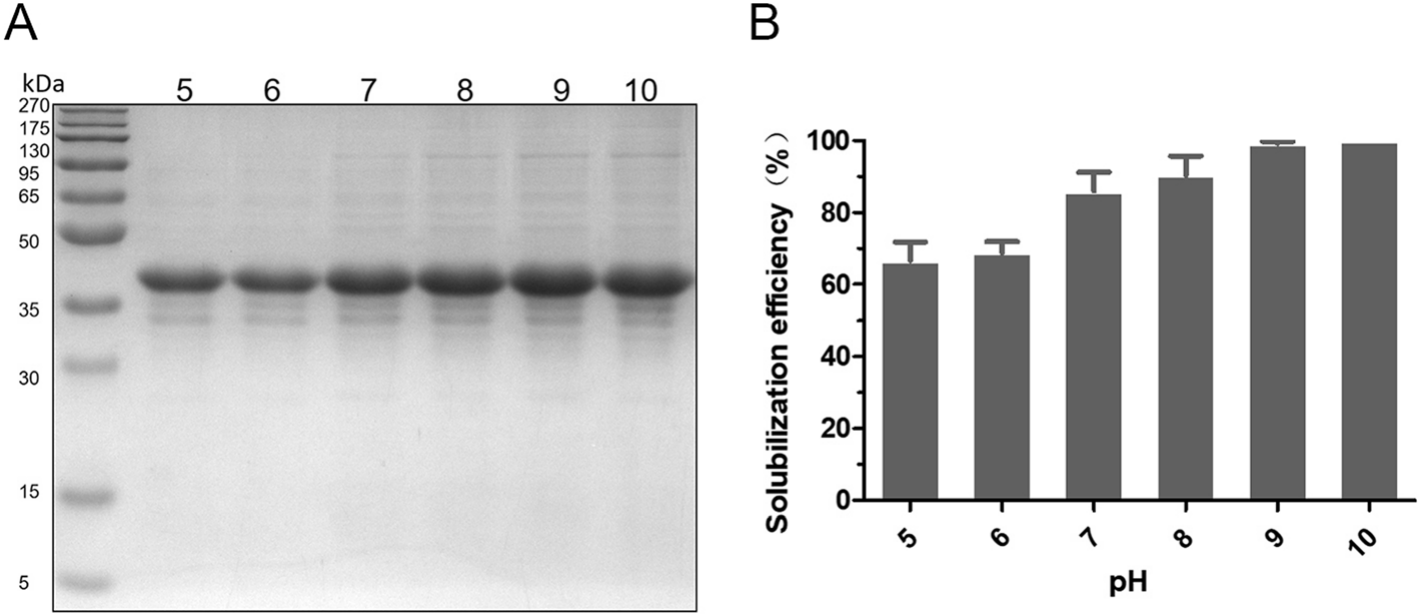
pH Effects on NM IBs solubilization with one-step heating method. The equal amount of purified NM IBs was suspended in 1 mL 10 mmol/L Tris-HCl at different pHs (range from 5.0 to 10.0) in the presence of 4 mol/L urea. **a** SDS-PAGE analysis of the effects of pH on NM IBs solubilization. **b** The quantification of solubilization efficiency at different pHs

In order to optimize the heating temperature that is essential for solubilization of NM IBs when performing one-step heating method, we tested effects from different temperatures, ranging from 40 to 100 °C, in 10 mmol/L Tris-HCl pH 8.0 containing 4 mol/L urea. As shown in Fig. 3, the solubilization capability was progressively increased when the heating temperature rose, and the plateau was reached at around 85 °C. Considering the thermal stability of spidroins, the heating temperature is optimized as 85 °C for following evaluations. Similarly, to find out the most suitable working pH the purified NM IBs were solubilized in 10 mmol/L Tris-HCl at different pHs (pH 5–10) containing 4 mol/L urea. The suspensions were mixed thoroughly and heated at 85 °C for 20 min. The soluble fractions containing NM were analyzed by SDS-PAGE. The results showed the solubilization efficiency was also affected by the working pH, with an increasing tendency from pH 5 to 7, while the different between pH 7, 8, 9 and 10 was not very obvious (Fig. 4), suggesting NM IBs solubilization by one-step heating method can be working under broad pH conditions.

### Solubilization of NM IBs in different buffers

**Fig. 5:**
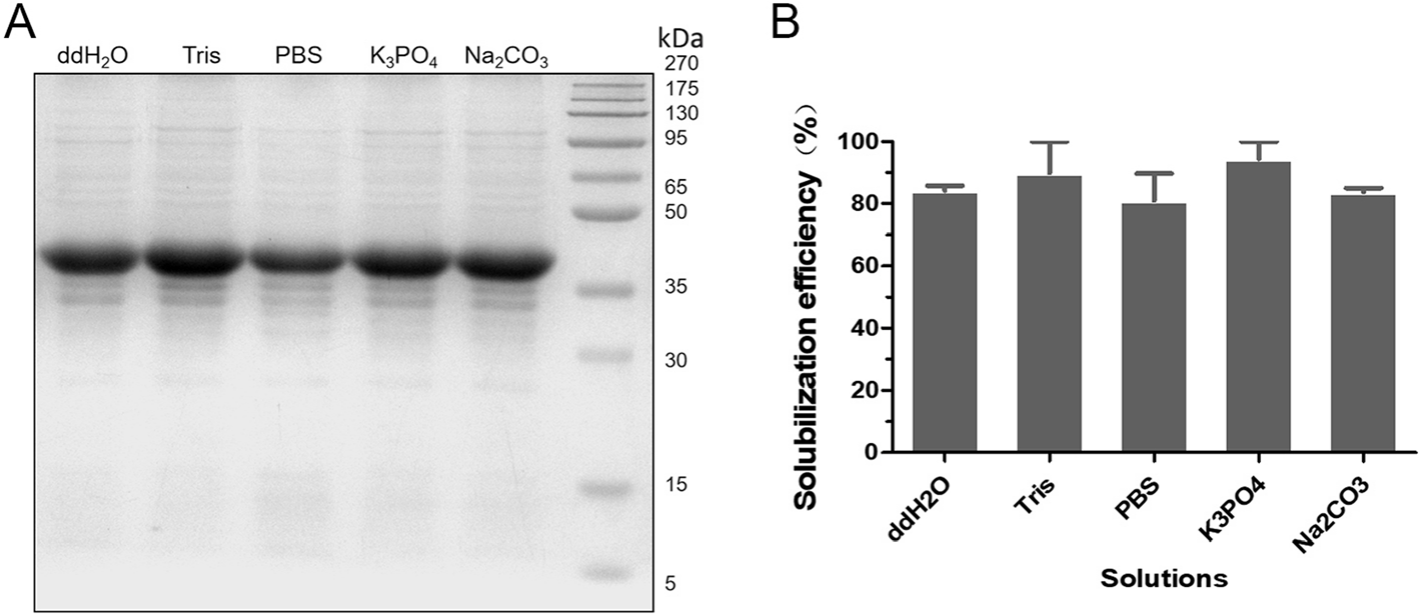
Effects of different buffers on the solubilization of NM IBs with one-step heating method. Equal amount of purified NM IBs was suspended in 1 mL different buffers containing 4 mol/L urea and solubilized by one-step heating method. **a** SDS-PAGE analysis of the effect of buffers on the solubility of NM IBs. ddH2O, deionized water; Tris, 10 mmol/L Tris–HCl pH 8.0; PBS, 1 × PBS; K_3_PO_4_, 10 mmol/L potassium phosphate pH 8.0; NaCO_3_, 10 mmol/L sodium carbonate pH 8.0. **b** The protein solubilization efficiency in different buffers

The solubilization of IBs might be influenced by different normally used buffer conditions. To figure this out, five different buffers were tested in this study. The results showed that the buffer conditions did not significantly affect the solubilization efficiency of NM IBs (Fig. 5a), indicated by the bands with similar intensity from 10 mmol/L NaCO_3_ (pH 8.0), 10 mmol/L K_3_PO_4_ (pH 8.0), 10 mmol/L Tris-HCl (pH 8.0) and deionized water (ddH2O), even though a slight decrease in 1 × PBS was observed. These results suggest the one-step heating method holds the potential to work under a wide range of biological buffers without significantly decreasing the solubilization capability.

### Self-assembly of NM protein into nanoparticles

**Fig. 6:**
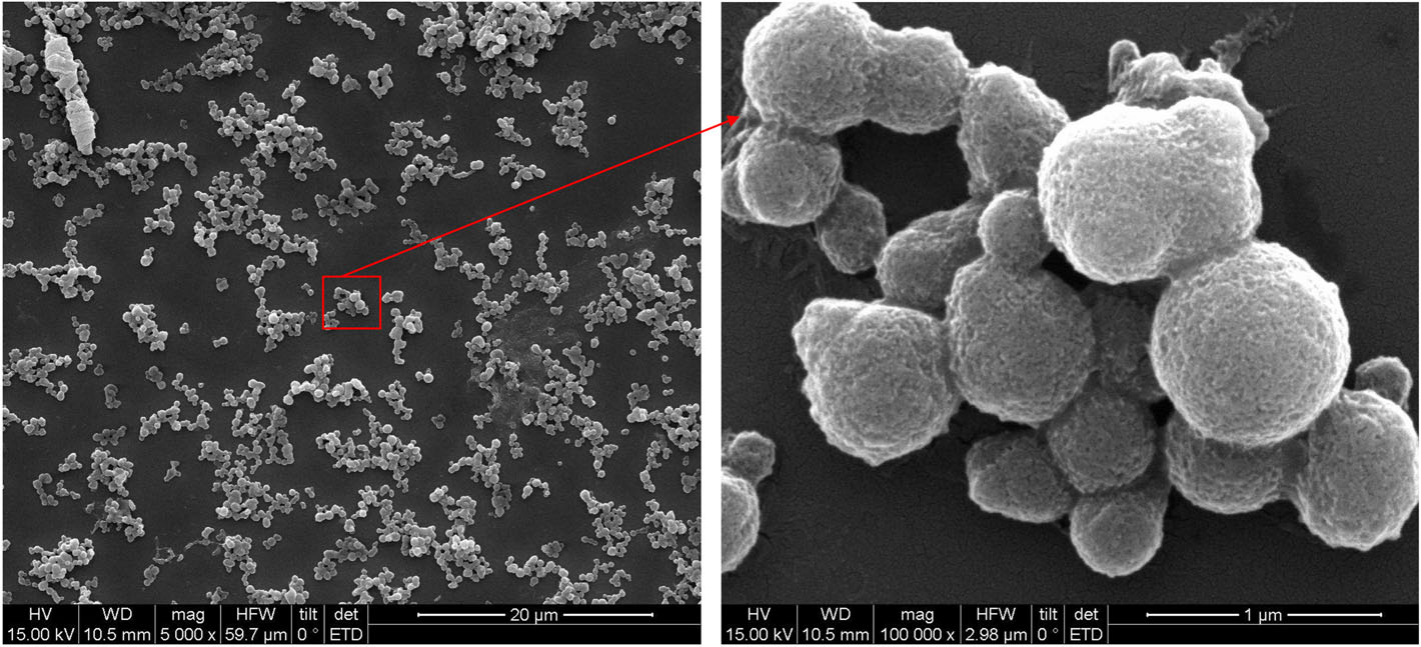
Characterization of NM nanoparticles. The NM nanoparticles formation was processed through salting out with potassium phosphate. The NM nanoparticles were completely washed with pure water and air-dried on a gold-coated silicon, and subsequently observed under Hitachi scanning electron microscope (Japan, S-4700) with HV as 15 kV. The nanoparticles were magnified 5000× (**a**) and 10,0000× (**b**) One hundred microliters NM solution (2.5 mg/mL) was mixed with 1 mL 2 M potassium phosphate buffer at a pH of 8.0 and was incubated at room temperature for 2 h.

To evaluate recombinant NM generated by the one-step heating method in a function point of view, the NM nanoparticle self-assembly was induced by salting out with potassium phosphate. As a result, the recombinant NM successfully self-assembled into nanoparticles. Under scanning electron microscope, well-distributed even spherical nanoparticles with diameter around 500 nm were observed (Fig. 6). The NM nanoparticles shared similar morphology as previous reported nanoparticles formed by other recombinant spidroins [2, 9], and might have potential and specific applications as functional biomaterials, e.g. controllable delivery of protein drugs /peptide vaccines.

### Identification of urea-induced modification

**Fig. 7:**
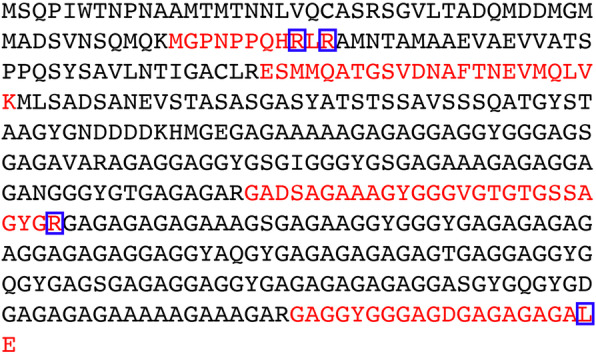
Carbamylation analysis of NM purified with the one-step heating protocol. The NM purified with the one-step heating protocol was trypsinated and analyzed by mass spectrometry. The peptide fragments observed by mass spectrometry were shown in red. The arginine and lysine that are carbamylated were boxed

To identify potential carbamylation from urea, the recombinant NM generated with above protocol was trypsinated and analyzed by mass spectrometry. Due to technique problems or the peptides their own properties, the peptides detected by mass spectrometry could not cover 100% of the full-length sequence; however, both oxidation and carbamylation were already observed (Supplementary Table 1). The cyanate group was detected on the sidechains of both Arg and Lys residues (Fig. 7, Supplementary Table 1). Although there is no evidence in this study to support the N terminal carbamylation of the recombinant NM solubilized with this heating protocol, still we cannot exclude this possibility. For the downstream experiments that are sensitive to carbamylation, the experiments should be designed carefully.

## Discussion

In current study, we introduce a one-step heating method, which enables efficient recovery of recombinant proteins, in particular spidroins with excellent purify from IBs. The recombinant spirdoins produced with this method can spontaneously self-assembled into structured spherical nanoparticles, which is similar to previously reported nanoparticles derived from other silk proteins. Further, the one-step heating method holds great compatibility to work under different biological buffers and pHs.

Over-expression of spidroins often forms IBs when recombinantly produced in *E. coli*. Normally, high concentration of denaturant such as 8 mol/L urea or 6 mol/L guanidine or organic solvents, e.g. hexafluoroisopropanol (HFIP) and formic acid, are used for a solubilization purpose. Subsequently, before processing into various formats extensive dialysis is applied to remove excess denaturant, leading to extra dilution and precipitation. To overcome this phenomenon, recently, many mild strategies for solubilizing IBs have been pursued for retaining native-like secondary structures, such as extreme pH, using detergents and freezing-thawing cycle [31, 33, 34]. Tag-free bioengineered spidroins were successfully prepared by thermal extraction and acidic extraction [27]; however, both methods are designed for soluble recombinant spidroins, while for spidroins in insoluble fraction high concentration denaturants are still necessary, which will break native-like secondary structures. Here, one-step heating method is a relatively mild solubilization strategy for dissolving engineered spidroins from IBs, with combining spidroins’ high thermal stability and the chaotropic properties of urea. With this method, the purity of solubilized NM from IBs was more than 95% without additional purification steps. The solubilization efficiency of NM IBs in Tris-HCl pH 8.0 containing 4 mol/L urea with this method was already comparable to that of 7 mol/L urea through traditional urea-denatured method. Even with 2 mol/L urea the solubilization efficiency of NM IBs was achieved 50% by one-step heating method, whereas there were only trace amounts of soluble protein obtained by traditional urea-denatured method (less than 5%) (Fig. 2). These results indicate that significantly less urea is required within one-step heating method, which confers great probability to maintain the secondary structures probably important for the downstream applications.

In terms of broad buffer and pHs conditions regarding various recombinant protein productions, the one-step heating method shows great feasibility. In current study, the solubilization efficiency of NM IBs was not very sensitive to normal buffer conditions, yet the different pHs and heating temperatures showed effects (Figs. 4 and 5). Although higher temperature promotes IBs solubilization, we do not exclude the possibility that the efficiency at relatively low temperature could be compensated by increasing incubation time. One-step heating method prefers neutral and alkaline conditions, however, under acidic condition it is still working but with slightly lower efficiency.

Despite the popularity as an effective protein denaturant, using urea increases the risk of carbamylation, in which the primary amino groups will be carbamylated [35]. The extent of carbamylation depends on the temperature, pH, and incubation time [36]. There are several ways to minimize protein/peptide carbamylation, e.g. always using freshly prepared urea solution from the highest-quality powder, avoiding long time heating, and selecting suitable buffers (like Tris-HCl, or ammonium containing buffers) [37]. In our protocol, the heating process is a prerequisite, but still to lower the risk getting carbamylated we recommend to use fresh urea with highest quality, Tris-HCl or or ammonium containing buffers, and shorten the incubation time. If the downstream application or testing is very sensitive to carbamylation, then this protocol should be optimized and applied carefully, otherwise other protocols should be pursued. Generally, we recommend the following conditions: heating temperature 70–90 °C for 20 min, pH 7.0–8.5, urea concentration 2–4 mol/L, and working buffer 10 mmol/L Tris-HCl or other ammonium containing buffers.

## Conclusions

The one-step heating method for solubilizing NM IBs is much more efficient and milder than the traditional urea-denatured method though urea-induced modifications may occur. Solubilization of NM IBs with low concentration of urea affords a good possibility for downstream nanoparticle or fibers formation. This method is not only suitable for recombinant spidroin preparation, but also holds the great potential for solubilizing other recombinant IBs forming proteins. If the downstream application of solubilized protein is sensitive to carbamylation, then this protocol should be applied carefully, due to the potential modification of carbamylation of amino groups in the existence of urea.

## Methods

### Expression and purification of NM IBs from *E. coli*

A gene fragment encoding a 430-aa protein (NM) that corresponds to the 261-aa repetitive sequence and the 161-aa NT domain of *A.ventricosus* MiSp was synthesized, and an enterokinase cleavage site (DDDDK) was introduced between the NT domain and the repetitive sequence (Fig. 1a). The gene fragment was inserted into pET-32a plasmid within *Nde*I and *Xho*I restriction sites (Fig. 1b) and confirmed by sequencing. The plasmid with correct sequence was transformed into *E. coli* BL21 (DE3) competent cells. For protein expression, the *E. coli* cells were grown at 37 °C in LB medium containing 30 μg/mL ampicillin until OD_600_ is around 1.0, and 1 mmol/L IPTG (final concentration) was added. The expression lasted for 4 h at 37 °C. The cells then were harvested by centrifugation and lysed by sonication for 30 min on ice. In order to obtain pure NM IBs, the insoluble pellets were washed as previously described [33].

### Solubilization of NM IBs

Equal amount of purified NM IBs was resuspended in Tris buffer at pH 8.0 containing different molar concentrations of urea (0–7 mol/L). For the traditional urea-denatured method, the suspension was stirred for 1 h at room temperature and centrifuged (12,000×g, 30 min, 4 °C) to collect the supernatant. For the one-step heating method, the suspension was heated at 85 °C for 20 min and centrifuged at 12,000×g for 30 min at 4 °C for collecting the supernatant. Supernatants were analyzed by SDS-PAGE. The soluble protein was quantified using Micro BCA Protein Assay Kit (Thermo, US). The solubilization efficiency from IBs was evaluated by the ratio of soluble protein concentration to that from 7 mol/L urea mediated one-step heating method.

### Solubilization of NM IBs in Tris buffer at different pHs and temperature

Equal amount of pure NM IBs was suspended in 1 mL 10 mmol/L Tris-HCl at different pHs (range from 5.0 to 10.0) in the presence of 4 mol/L urea. The homogenous IBs suspensions were heated at 85 °C for 20 min and centrifuged at 12,000×g for 30 min at 4 °C for supernatant collections. On the other hand, homogenous suspensions of NM IBs were suspended in 10 mmol/L Tris-HCl at pH 8.0 containing 4 mol/L urea. The suspensions were heated at different temperatures (40–100 °C) for 20 min and centrifuged. Supernatants were analyzed by SDS-PAGE and protein concentrations were quantified using Micro BCA Protein Assay Kit. The protein solubilization efficiency was evaluated by the ratio of soluble protein to total protein.

### Solubility of NM IBs in different buffers

Five different biological buffers in presence of 4 mol/L urea were employed to solubilize NM IBs: 1 × PBS, 10 mmol/L sodium carbonate (NaCO_3_, pH 8.0), 10 mmol/L potassium phosphate (K_3_PO_4_, pH 8.0), 10 mmol/L Tris–HCl (pH 8.0), and deionized water (ddH2O). Equal amount of purified NM IBs was suspended in 1 mL different buffers and mixed thoroughly to get homogenous suspensions. The IBs suspensions were heated at 85 °C for 20 min and centrifuged at 12,000 g for 30 min at 4 °C for clear supernatants collection, respectively. Supernatants were analyzed parallelly by SDS-PAGE and the protein solubilization efficiency was evaluated as above.

### Preparation of NM nanoparticles

The recombinant NM from IBs prepared with one-step heating method was dialyzed in 10 mmol/L Tris-HCl at pH 8.0. One hundred microliters NM solution (2.5 mg/mL) was mixed with 1 mL 2 mol/L potassium phosphate buffer at a pH of 8.0. The solution was incubated at room temperature for 2 h and then dialyzed overnight against ultrapure water. For scanning electron microscopy (SEM), NM nanoparticles were completely washed with pure water and air-dried on a gold-coated silicon. The particles were observed under a Hitachi scanning electron microscope (Japan, S-4700).

### Mass spectrometry analysis

NM protein solubilized from IBs generated by one-step heating method was analyzed with nano-LC mass spectrometry on an EASY-nLC II (Thermo Fisher Scientific). The sample was digested with trypsin (Promega) at 37 °C for 20 h. The digested mix was lyophilized and resuspended in 0.1% FA. For each full scan Map (MS2 scan), twenty fragments were collected. The raw data were processed using Mascot 2.2 program, and potential modifications, e.g. carbamylation and oxidation were analyzed.

## Supplementary information

**Additional file 1.**

## Data Availability

All data generated or analyzed during this study are included in this published article.

## Abbreviations

NM IBs: NM inclusion bodies
MaSp: Major ampullate spidroin
MiSp: Minor ampullate spidroin
*E. coli*: *Escherichia coli*
*A. ventricosus*: *Araneus ventricosus*
NT: N-terminal domain
CT: C-terminal domain
SEM: Scanning electron microscopy
